# Advances in diagnosis, management, and long-term outcomes of pediatric vasovagal syncope: a comprehensive review

**DOI:** 10.3389/fcvm.2025.1481749

**Published:** 2025-04-25

**Authors:** Wenjing Zhu, Xueyan Bian, Jianli Lv

**Affiliations:** ^1^ Department of Pulmonary and Critical Care Medicine, Shandong Provincial Hospital Affiliated to Shandong First Medical University, Shandong Provincial Clinical Research Center for Children’s Health and Disease Office, Jinan, Shandong, China; ^2^ Department of Pediatrics, Lixia District People’s Hospital, Jinan, Shandong, China; ^3^ Department of Pediatric Cardiology, Shandong Provincial Hospital Affiliated to Shandong First Medical University, Shandong Provincial Clinical Research Center for Children’s Health and Disease Office, Jinan, Shandong, China

**Keywords:** vasovagal syncope, pediatric, diagnosis, management, outcomes

## Abstract

Vasovagal syncope (VVS) is the most common cause of transient loss of consciousness in children and adolescents, accounting for 60%–80% of syncope cases. This review synthesizes current evidence on pediatric VVS, focusing on advances in diagnosis, management, and long-term outcomes. Through a comprehensive literature search of studies published between 2001 and 2024, we analyzed epidemiological patterns, pathophysiological mechanisms, diagnostic approaches, management strategies, and prognostic factors. Recent diagnostic advances include implantable loop recorders and smartphone applications, which have improved diagnostic accuracy. Management has evolved toward individualized approaches, emphasizing non-pharmacological interventions (hydration, salt supplementation, physical counterpressure maneuvers) as first-line treatment, with medications such as midodrine and fludrocortisone showing variable efficacy in refractory cases. Long-term studies indicate that while most children experience improvement over time, 33%–50% have recurrent episodes within three years, with factors such as lower mean arterial pressure, higher urine specific gravity, younger age, family history of syncope, and lower body mass index associated with increased recurrence risk. Though generally benign, VVS can significantly impact quality of life and carries substantial psychosocial consequences. Future research should focus on developing predictive models for recurrence risk and exploring personalized treatment approaches to improve outcomes.

## 1 Introduction

Vasovagal syncope (VVS) represents the predominant etiology of transient loss of consciousness in pediatric and adolescent populations, accounting for approximately 60%–80% of syncope cases (1–3). Characterized by an abrupt, temporary loss of consciousness resulting from global cerebral hypoperfusion, VVS manifests as a consequence of an exaggerated autonomic response to various triggers, precipitating bradycardia and/or peripheral vasodilation (4).

Despite its prevalence, the diagnosis and management of pediatric VVS present significant challenges. The clinical presentation varies considerably, and the differential diagnosis encompasses a spectrum of conditions from benign neurocardiogenic syndromes to potentially life-threatening cardiac conditions (5). Recent advances in diagnostic modalities and management strategies have improved outcomes, yet significant knowledge gaps persist regarding long-term prognosis and optimal treatment approaches for refractory cases (6, 7).

This review aims to synthesize current evidence on pediatric VVS, highlighting recent advances in diagnosis and management while identifying knowledge gaps that should guide future research.

## 2 Methods

We conducted a comprehensive literature search following the Preferred Reporting Items for Systematic Reviews and Meta-Analyses (PRISMA) guidelines. Electronic databases including PubMed/MEDLINE, EMBASE, and the Cochrane Library were searched for articles published between January 2001 and March 2024. The search strategy employed keywords and Medical Subject Headings (MeSH) terms including “vasovagal syncope,” “neurocardiogenic syncope,” “pediatric,” “children,” “adolescents,” “diagnosis,” “management,” and “outcomes” in various combinations.

Inclusion criteria were: (1) studies focusing on VVS in pediatric populations (age ≤18 years); (2) publications in English; (3) studies addressing aspects of diagnosis, management, or outcomes. We prioritized original research, systematic reviews, meta-analyses, and evidence-based clinical guidelines. After initial screening titles and abstracts, relevant articles underwent full-text review by two independent reviewers, with disagreements resolved through consensus discussion.

Due to the heterogeneity of study designs and outcome measures, we employed a narrative synthesis approach, organizing evidence according to key themes including epidemiology, pathophysiology, diagnosis, management, and long-term outcomes, with emphasis on pediatric-specific considerations and recent advances.

## 3 Results

### 3.1 Literature search results

**Figure 1:**
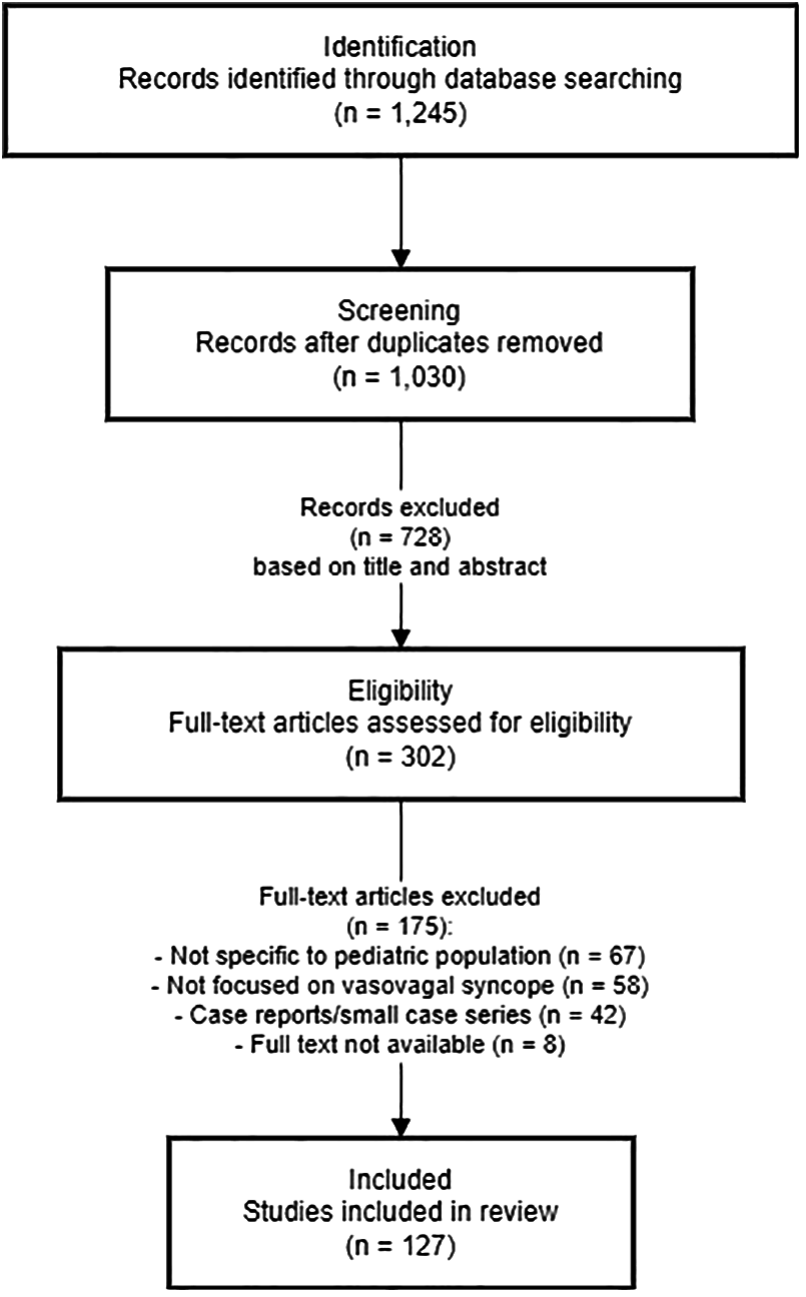
Flow diagram of literature search and selection process.

The literature search process and results are summarized in Figure 1. Initial database searches yielded 1,245 potentially relevant citations. After removing duplicates (*n* = 215), 1,030 articles were screened based on titles and abstracts. Of these, 728 were excluded for not meeting inclusion criteria. The remaining 302 articles underwent full-text review, resulting in 175 articles being excluded for various reasons (not specific to pediatric population, not focused on VVS, or being case reports with limited generalizability). Ultimately, 127 articles were included in this comprehensive review.

### 3.2 Epidemiological patterns of pediatric VVS

Our analysis of epidemiological studies revealed significant patterns in the prevalence and distribution of VVS in children and adolescents. VVS accounts for 60%–80% of syncope cases in pediatric populations (1–3), with syncope representing 1%–3% of emergency department visits and having an overall incidence of 0.1%–0.5% in the pediatric population (8).

Age-specific patterns show that the incidence of VVS increases dramatically during adolescence, peaking between ages 15–19 years (2). Population-based studies demonstrate a notable gender disparity, with a cumulative incidence of 41% for females and 28% for males (9). Female sex is consistently identified as a significant risk factor, with girls being 1.5–2 times more likely to experience VVS than boys (10, 11).

Additional predisposing factors include family history of syncope, low body mass index, anxiety disorders and orthostatic intolerance (12, 13). Environmental triggers such as dehydration, prolonged standing, and exposure to hot environments are commonly identified (4), while a sedentary lifestyle and poor physical conditioning may increase susceptibility (14).

The recurrence rate of VVS in children and adolescents is substantial. Studies report that 33%–50% of patients experience at least one recurrent episode within three years of the initial event (15). However, the frequency and severity of episodes tend to decrease over time, with many patients experiencing improvement by early adulthood (16).

Despite its generally benign nature, VVS carries a significant economic burden, estimated at several hundred million dollars annually in the United States (17), reflecting costs associated with emergency department visits, hospitalizations, and diagnostic evaluations.

### 3.3 Pathophysiological mechanisms underlying pediatric VVS

The pathophysiology of VVS involves complex interactions between cardiovascular, neurological, and neuroendocrine systems. The fundamental process involves a sudden, inappropriate decrease in systemic blood pressure and/or heart rate, leading to transient global cerebral hypoperfusion (18).

The classic model centers on the Bezold-Jarisch reflex, a cardio-inhibitory response triggered by mechanoreceptors (C-fibers) in the left ventricle (19). In susceptible individuals, various stimuli such as prolonged standing or emotional stress lead to excessive venous pooling and reduced venous return. This results in vigorous contraction of an underfilled left ventricle, activating mechanoreceptors and initiating the reflex (18).

The activated reflex causes paradoxical bradycardia and vasodilation through increased parasympathetic activity and withdrawal of sympathetic tone (20). This response is mediated by the nucleus tractus solitarius in the brainstem, which receives afferent signals from mechanoreceptors and modulates efferent autonomic outflow (21).

Recent research has highlighted the role of impaired baroreflex sensitivity in VVS. Studies have demonstrated that children with recurrent VVS exhibit reduced baroreflex sensitivity compared to healthy controls, suggesting dysfunction in blood pressure homeostasis (22).

Neuroendocrine factors also contribute to VVS pathophysiology. Elevated levels of epinephrine have been observed during syncopal episodes, potentially exacerbating vasodilation (23). Alterations in serotonergic neurotransmission have been implicated in modulating central autonomic responses (24).

Genetic factors may predispose certain individuals to VVS, with familial clustering observed and potential genetic loci identified (13). Age-related differences in autonomic function, particularly during puberty, may explain the increased prevalence in adolescents. Puberty is associated with significant changes in cardiovascular autonomic regulation, including alterations in baroreflex sensitivity and sympathovagal balance (25).

### 3.4 Advances in diagnostic approaches for pediatric VVS

The diagnosis of VVS in children requires a comprehensive approach, combining careful history-taking, physical examination, and selective use of diagnostic tests. The primary goal is to differentiate VVS from other potentially life-threatening causes of syncope (26).

A detailed clinical history remains the cornerstone of VVS diagnosis. Key elements include identifying precipitating factors such as prolonged standing or emotional stress (4); recognizing prodromal symptoms like lightheadedness, nausea, and visual changes (27); characterizing the event, including duration and rapid recovery (28); and assessing post-syncopal such as fatigue and nausea (29).

Physical examination should include comprehensive cardiovascular assessment (heart rate, blood pressure, orthostatic measurements) and neurological examination to exclude other etiologies (5).

While diagnosis is often based on clinical presentation, several diagnostic tests may be employed. A 12-lead ECG is recommended for all children presenting with syncope to rule out cardiac causes such as long QT syndrome or Brugada syndrome (2). Echocardiography may be indicated when structural heart disease is suspected based on history, examination, or ECG findings (30).

The Head-Up Tilt Table Test (HUTT) is considered the gold standard for diagnosing VVS, with a sensitivity of 60%–70% and specificity of 90%–95% in pediatric populations (31, 32). The test involves passive standing on a tilt table at a 60–70 degree angle for up to 45 min, with continuous monitoring of heart rate and blood pressure. A positive test is defined as the reproduction of syncope or pre-syncope associated with hypotension and/or bradycardia (33).

Recent technological advances have expanded the diagnostic arsenal. Implantable loop recorders (ILRs) allow for long-term cardiac monitoring in cases of recurrent, unexplained syncope. A study by Placidi et al. demonstrated the effectiveness of miniaturized ILRs in pediatric patients, enabling correlation of symptoms with cardiac rhythm (34). Exercise stress testing may be useful in cases of exertion-related syncope (35).

Smartphone-based ECG applications represent another significant advancement, providing non-invasive, cost-effective means of arrhythmia detection (36). These technologies facilitate ambulatory monitoring and may improve diagnostic yield in cases where conventional monitoring is inconclusive.

Diagnostic challenges in pediatric VVS include variability in presentation across different age groups (37), difficulty obtaining clear history from younger children (38), and symptom overlap with other conditions, particularly anxiety disorders (39). Careful consideration of differential diagnoses, including cardiac syncope, neurological causes such as seizures, orthostatic hypotension, and psychogenic pseudosyncope, is essential (40, 41).

### 3.5 Evolution of management strategies for pediatric VVS

Management of VVS in pediatric patients has evolved toward a multifaceted, individualized approach based on clinical presentation, episode frequency, and impact on quality of life (26, 42).

Non-pharmacological interventions form the cornerstone of VVS management. Comprehensive patient and family education is essential, focusing on trigger identification and avoidance (43). Optimizing hydration (>2 L/day) and salt intake (>6 g/day) has shown effectiveness, particularly in patients with orthostatic intolerance (44). Physical counterpressure maneuvers, involving isometric muscle contractions to augment venous return during prodromal symptoms, have demonstrated efficacy in reducing syncope recurrence (45).

Structured exercise programs focusing on aerobic conditioning and lower limb strength training have shown promise. A randomized controlled trial reported that yoga as an adjunctive therapy significantly reduced syncopal and presyncopal events and improved quality of life compared to standard therapy alone (46). Orthostatic training (tilt training), involving progressively prolonged periods of upright posture, has also demonstrated effectiveness. Clinical experience suggests that these interventions pose no harm and may improve outcomes and reduce the frequency of episodes (47).

Pharmacological interventions are typically reserved for patients with frequent, severe episodes or those refractory to non-pharmacological measures. Fludrocortisone, a mineralocorticoid promoting sodium and water retention, has been studied in children with VVS. However, the multicenter Prevention of Syncope Trial (POST) 2 did not meet its primary objective of demonstrating significant reduction in syncope recurrence compared to placebo (48).

Midodrine, an α1-adrenergic agonist increasing peripheral vascular resistance, has shown more promise. A randomized controlled study in children with VVS demonstrated that the addition of midodrine hydrochloride to conventional therapy significantly reduced syncope recurrence rates compared to conventional therapy alone, with a significantly lower recurrence rate during follow-up periods of at least 6 months (49). Beta-blockers, while commonly prescribed, have demonstrated limited efficacy. A randomized controlled trial comparing metoprolol to conventional treatment in children and adolescents found no significant difference in syncope recurrence (50).

Selective serotonin reuptake inhibitors (SSRIs) may modulate central autonomic responses (51), but pediatric data are limited. Invasive interventions, including cardiac pacing with rate-drop response algorithms and catheter ablation of ganglionated plexus (52–54), are rarely indicated in pediatric VVS and reserved for severe, refractory cases (55).

A stepwise approach to management is recommended, beginning with education and lifestyle modifications, progressing to structured exercise programs, and considering pharmacological therapy for frequent or severe cases.

Regular follow-up is essential to assess treatment efficacy and adjust management strategies. Objective measures, such as the Calgary Syncope Symptom Score or quality of life assessments, can be utilized to quantify clinical improvement (56).

Special considerations in pediatric VVS management include addressing the psychological burden through cognitive-behavioral therapy or other psychological interventions (57), and individualized risk stratification for young athletes (58).

Several novel approaches are under investigation for refractory VVS, such as ivabradine (a selective If channel inhibitor) (59). Given the benign nature of VVS, cost-effectiveness should be a consideration in management decisions, with a conservative approach being most cost-effective for initial management (60).

### 3.6 Long-term outcomes and prognostic factors in pediatric VVS

The prognosis of VVS in children is generally favorable, with most patients experiencing improvement or resolution of symptoms over time. However, natural history and long-term outcomes vary significantly among individuals.

The natural course of pediatric VVS tends toward spontaneous improvement. A study of 29 pediatric patients with neurocardiogenic syncope found that clinical events were greatly reduced in both treated and untreated groups during follow-up, with recurrences becoming unlikely after 24 months (61). This improvement is thought to be related to physiological maturation of the autonomic nervous system and adaptation to orthostatic stress.

Despite the overall favorable prognosis, recurrence rates remain substantial. In a study of 352 children with VVS followed for a median of 22 months, factors associated with an increased risk of recurrence included lower mean arterial pressure in the supine position, higher urine specific gravity, younger age, family history of syncope, and lower body mass index (62). The study developed a prognostic nomogram model incorporating these factors, which showed good predictive ability for 1-year, 2-year, and 3-year recurrence rates.

While not life-threatening, VVS can significantly impact quality of life. A cross-sectional study using the PedsQL™ 4.0 Generic Core Scales found that children with recurrent VVS had lower scores in physical, emotional, and social functioning compared to healthy controls (63). However, with appropriate management and patient education, most children can achieve good symptom control and maintain normal activities (64).

There is no evidence suggesting that childhood VVS is associated with increased cardiovascular morbidity or mortality in adulthood. A large population-based cohort study with a median follow-up of 17 years found no increased risk of major adverse cardiovascular events in individuals with VVS history compared to the general population (65).

The psychological impact of VVS in children and adolescents should not be underestimated. A prospective case-control study found significantly higher rates of psychopathology in VVS patients compared to controls, with 21.3% meeting criteria for major depressive disorder (vs. 2% of controls) and 19.1% diagnosed with social anxiety disorder, generalized anxiety disorder, or conversion disorder (57). Early intervention and psychological support may mitigate these long-term psychosocial effects.

Several factors predict long-term outcomes in pediatric VVS. A higher number of syncopal episodes prior to diagnosis predicts a more protracted course (15, 66), while early improvement with conservative measures suggests favorable long-term outcomes (45).

For young athletes with VVS, prognosis is generally good with appropriate management. Many are able to return to competitive activities after proper diagnosis and treatment (67). Children with a predominantly cardioinhibitory response on tilt testing may have higher risk of recurrent syncope and injury (68), but long-term studies have not demonstrated increased risk of sudden cardiac death in this subgroup (65).

## 4 Discussion

This comprehensive review highlights significant advances in understanding and managing pediatric VVS, while also identifying persistent challenges and knowledge gaps. Our findings underscore the multifaceted nature of VVS in children and adolescents, requiring individualized diagnostic and management approaches.

The epidemiological data presented confirm that VVS represents a significant health burden in pediatric populations, with a notable peak during adolescence and higher prevalence in females. This gender disparity warrants further investigation into potential hormonal and physiological factors that may contribute to increased susceptibility in girls.

Recent diagnostic advances, including implantable loop recorders and smartphone-based ECG applications, have expanded the toolkit for clinicians evaluating syncope in children. However, these technologies should complement rather than replace thorough clinical assessment. The traditional approach of detailed history-taking and physical examination remains the cornerstone of diagnosis, with technological adjuncts providing valuable confirmatory evidence.

Our review of management strategies reveals a paradigm shift toward non-pharmacological interventions as first-line therapy. The demonstrated efficacy of hydration, salt supplementation, physical counterpressure maneuvers, and structured exercise programs supports a conservative initial approach. The variable efficacy of pharmacological options suggests the need for improved patient selection criteria to identify those most likely to benefit from medication.

The long-term outcome data present a generally reassuring picture, with most children experiencing improvement over time. However, the substantial recurrence rate (33%–50% within three years) and identified risk factors for recurrence highlight the need for prognostic tools to guide monitoring and management intensity. The development of validated prediction models, such as the nomogram described by Sun et al. (62), represents a promising step toward personalized risk stratification.

Several important limitations in current evidence merit consideration. First, many studies have relatively short follow-up periods, limiting understanding of very long-term outcomes extending into adulthood. Second, heterogeneity in outcome measures across studies complicates direct comparison of intervention efficacy. Third, most studies have not addressed the impact of comorbidities, particularly psychological conditions, on VVS presentation and management.

Future research should focus on several key areas. Genetic and molecular studies may identify specific markers for VVS susceptibility, enabling targeted therapies. Longitudinal studies with extended follow-up are needed to elucidate the natural history into adulthood. Randomized controlled trials of emerging therapies, particularly in refractory cases, would address significant treatment gaps. Finally, standardization of outcome measures across studies would facilitate meta-analysis and strengthen evidence-based recommendations.

From a clinical perspective, our findings support a stepwise, individualized approach to VVS management, beginning with education and lifestyle modifications before considering pharmacological interventions. The significant psychosocial impact of VVS underscores the importance of addressing both physical and psychological aspects of the condition. Multidisciplinary collaboration among cardiologists, neurologists, psychologists, and primary care providers would optimize comprehensive care.

## 5 Conclusion

This review synthesizes current evidence on pediatric VVS, highlighting advances in diagnosis, management, and understanding of long-term outcomes. While technological innovations have improved diagnostic capabilities, and evidence increasingly supports non-pharmacological interventions as first-line therapy, significant challenges remain in predicting individual outcomes and managing refractory cases.

VVS in children represents a complex clinical entity requiring individualized assessment and management. Although generally benign with favorable long-term prognosis, its impact on quality of life and potential psychological consequences necessitate comprehensive care approaches.

Future research should focus on developing robust prediction models for recurrence risk, evaluating emerging therapeutic options, and establishing standardized outcome measures to strengthen the evidence base. By bridging the gap between scientific advances and clinical practice, we can continue to improve outcomes for the many children and adolescents affected by VVS.
